# A rare case of acquired von Willebrand syndrome type 2B: diagnosis, treatment, and underlying pathophysiology

**DOI:** 10.1016/j.rpth.2024.102516

**Published:** 2024-07-19

**Authors:** Simon Jaouen, Fanny Mingant, Brigitte Pan-Petesch, Eric Lippert, Emmanuelle Jeanpierre, Hubert Galinat

**Affiliations:** 1 Hematology Laboratory, Brest University Hospital, Brest, France; 2 Department of Hematology, Brest University Hospital, Brest, France; 3 Hemostasis and Transfusion Department, Lille University Hospital, Lille, France

**Keywords:** acquired von Willebrand syndrome, hemorrhage, monoclonal gammopathy, platelet glycoprotein GPIb, type 2B von Willebrand disease

## Abstract

**Background:**

Acquired von Willebrand syndrome (AVWS) is a rare bleeding disorder that usually mimics type 1 or 2A von Willebrand disease (VWD).

**Key Clinical Question:**

Can AVWS mimic the phenotype of type 2B VWD?

**Clinical Approach:**

A 64-year-old male patient presented with thrombocytopenia, normal routine hemostasis results, and normal VWF antigen and factor VIII levels but reduced von Willebrand factor (VWF) activity (31 IU/dL). The ristocetin-induced platelet aggregation test showed paradoxical aggregation at low doses of ristocetin, suggesting type 2B VWD, but no deleterious sequence variation was found in either the *VWF* or *GP1BA* genes, compatible with AVWS. Serum protein electrophoresis revealed a monoclonal immunoglobulin G antibody.

**Conclusion:**

This AVWS with a 2B phenotype VWD was probably related to a monoclonal immunoglobulin G antibody causing a VWF conformational change, resulting in increased affinity to platelet glycoprotein-Ib. In the event of surgery or bleeding, treatment with vonicog alfa seems to be the best option for this patient.

## Essentials

• Acquired von Willebrand syndrome can mimic type 2B von Willebrand disease.
• A patient with thrombocytopenia and bleeding symptoms was required to undergo a prostate biopsy.
• Monoclonal immunoglobulin G causes von Willebrand factor conformational change, resulting in type 2B acquired von Willebrand syndrome.
• Recombinant von Willebrand factor concentrate has been shown to be more effective than intravenous immunoglobulin.

## 1 Introduction

Acquired von Willebrand syndrome (AVWS) is a rare bleeding disorder, the diagnosis of which can be challenging. The main etiologies of AVWS include lymphoproliferative syndromes [1], myeloproliferative syndromes [2], cardiovascular diseases [[3], [4], [5]], autoimmune diseases, and hypothyroidism [6,7]. The primary mechanisms involved in AVWS include defect in von Willebrand factor (VWF) synthesis, increased VWF proteolysis, mechanical destruction of VWF, neutralizing or nonneutralizing autoantibodies resulting in decreased VWF activity or increased VWF clearance, and tumor cellular adsorption of VWF multimers [8]. AVWS usually mimics type 1 or 2A von Willebrand disease (VWD). The primary treatment of AVWS depends on its etiology. Nevertheless, prophylactic or therapeutic measures to control bleeding may be necessary in some cases. Despite its short-lived effects [9], desmopressin remains the most commonly used treatment for AVWS. Other strategies include administration of intravenous immunoglobulin (IVIG), VWF, or recombinant-activated factor (F)VII (rFVIIa) [[10], [11], [12], [13]]. The following case report addresses the question of whether AVWS can also mimic a type 2B VWD. The literature reports only 2 clinical cases of type 2B AVWS. The use of desmopressin is generally not recommended for patients with type 2B VWD (VWD2B) due to the high risk of severe thrombocytopenia. This condition may necessitate careful assessment of various treatment options.

## 2 Case Report

We report herein the case of a 64-year-old man who was referred to the Brest University Hospital for reevaluation of thrombocytopenia before a prostate biopsy. This study is in accordance with the Helsinki Declaration, and the participant gave written informed consent.

The patient had a 10-year history of isolated and chronic fluctuating thrombocytopenia (50-80 × 10^9^/L), with occasional platelet aggregates. Immune thrombocytopenic purpura was initially diagnosed despite resistance to corticosteroids or IVIG. The patient underwent 3 surgeries without bleeding complications but has experienced unusual bruising since 2007 associated with moderate thrombocytopenia. There was no family history of thrombocytopenia or bleeding.

**Figure 1:**
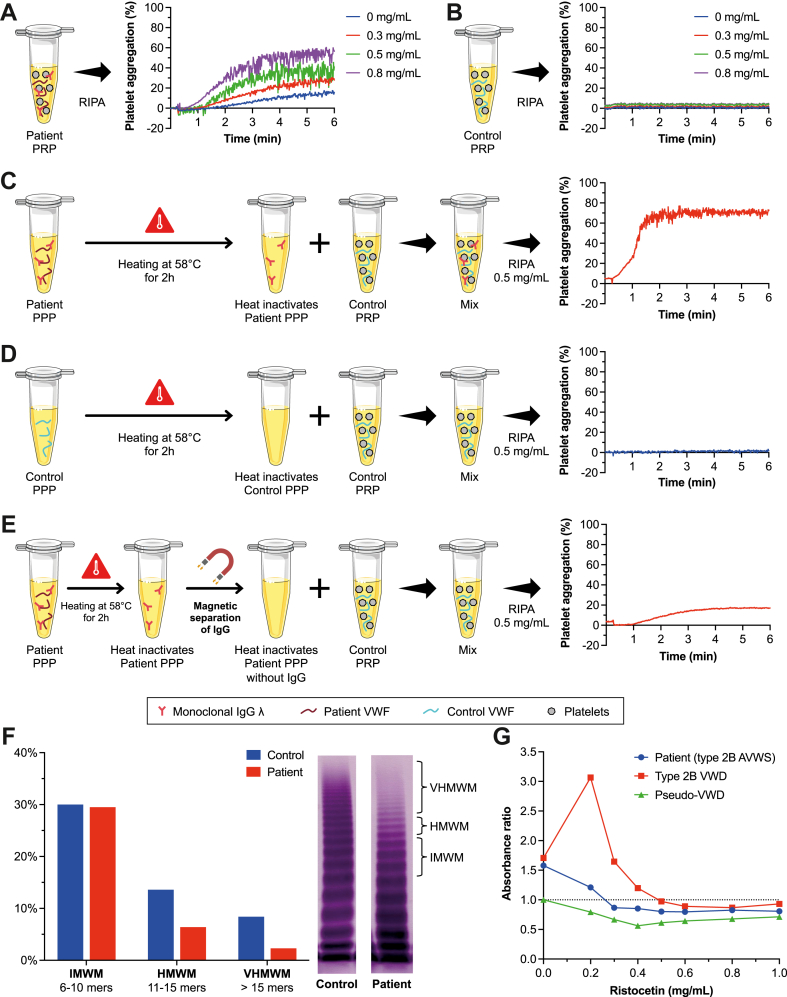
**Monoclonal immunoglobulin (Ig)G involvement in the acquired von Willebrand syndrome (AVWS).** (A and B) Ristocetin-induced platelet aggregation (RIPA) at low doses of ristocetin (0, 0.3, 0.5, and 0.8 mg/mL) with patient platelet-rich plasma (PRP; A) and control PRP (B). (C and D) RIPA was performed at 0.5 mg/mL on a mixture (1:1 ratio) of control PRP with heated (58 °C for 2 hours) patient platelet-poor plasma (PPP; C) and heated (58 °C for 2 hours) control PPP (D). (E) RIPA was performed at 0.5 mg/mL on a mixture (1:1 ratio) of control PRP and heated (58 °C for 2 hours) IgG-depleted (anti-Human IgG magnetic beads) patient PPP. (F) Distribution of von Willebrand factor (VWF) multimers (gel electrophoresis) for control (Standard Human Plasma, Siemens Healthineers) and patient samples. (G) VWF binding to recombinant glycoprotein (GP)-Ibα for different ristocetin concentrations—enzyme-linked immunoassay absorbance ratios (patient/control) are at 490 nm; our patient type 2B AVWS (blue circles), constitutional 2B von Willebrand disease (VWD; red squares), and pseudo-VWD (green triangles). The dotted line represents an absorbance ratio of 1. HMWM, high-molecular-weight multimers (11-15 mers); IMWM, intermediate-molecular-weight multimers (6-10 mers); VHMWM, very high-molecular-weight multimers (>15 mers).

Two monoclonal immunoglobulin (Ig)G lambda peaks were observed on the serum protein electrophoresis, quantified at 6 g/L (Supplementary Figure). Blood counts were normal except for severe thrombocytopenia at 42 × 10^9^/L. Prothrombin time, activated partial thromboplastin time, and fibrinogen levels were normal. FVIII and VWF antigen (VWF:Ag) levels were 152 and 111 IU/dL, respectively. However, VWF activity (VWF:Ac) was reduced to 31 IU/dL using VWF:GPIbM method (Innovance VWF:Ac, Siemens; VWF:GPIbM/VWF:Ag ratio = 0.28) and to 51 IU/dL using VWF:GPIbR method (HemosIL ACUSTAR VWF:RCo, Werfen; VWF:GPIbR/VWF:Ag ratio = 0.44). Ristocetin-induced platelet aggregation (RIPA) revealed a paradoxical aggregation at low doses of ristocetin (<0.5 mg/mL) with patient plasma compared with healthy donor control plasma (Figure 1A, B). Nevertheless, the *VWF* and *GP1BA* genes showed no deleterious sequence variations.

After heating patient platelet-poor plasma (PPP) at 58°C for 2 hours to inactivate VWF and other factors while sparing immunoglobulins and mixing it with control platelet-rich plasma (PRP), RIPA was positive at low doses, but this was not the case when mixing the heated control PPP with control PRP (Figure 1C, D). In addition, when the heated patient PPP was depleted of IgG using anti-Human IgG Magnetic Beads (RayBiotech) and mixed with a control PPP, RIPA was significantly reduced (Figure 1E).

Distribution of VWF multimers on gel electrophoresis revealed a loss of the high-molecular-weight (HMW) forms in the patient (Figure 1F). Enzyme-linked immunoassay ristocetin-induced binding of VWF to recombinant fragment of glycoprotein (GP)-Ibα did not show hyperfixation at the low ristocetin concentration of 0.2 mg/mL, unlike constitutional VWD2B (Figure 1G). Enzyme-linked immunoassay anti-VWF assay revealed the presence of a low-titer anti-VWF (1.5 times the positivity cutoff: optical density = 0.296 for a positivity cutoff of 0.200), and VWF:propeptide (pp)/VWF:Ag ratio was normal at 1.38.

**Figure 2:**
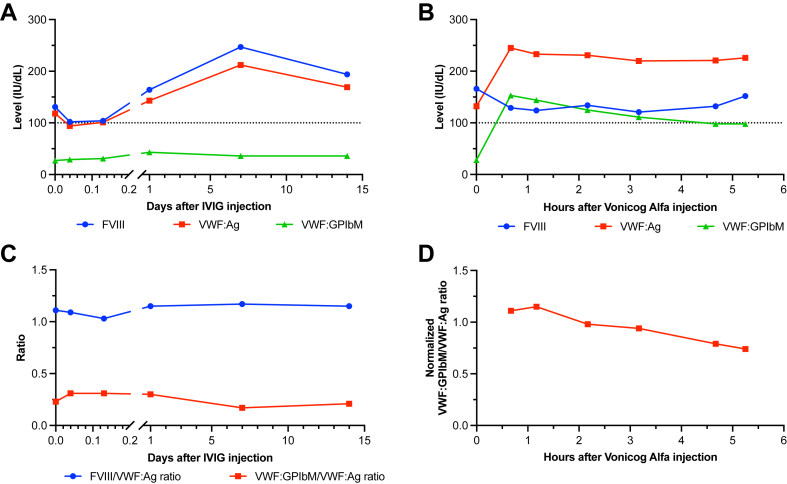
**Treatment responses.** (A) Level of factor (F)VIII activity, von Willebrand factor (VWF) antigen (VWF:Ag), and VWF activity (VWF:GPIbM) at 0 (t_0_), 0.04, 0.13, 1, 7, and 14 days after intravenous immunoglobulin (IVIG) injection (1 g/kg). (B) Level of FVIII, VWF:Ag, and VWF:GPIbM at 0 (t_0_), 0.67, 1.17, 2.17, 3.17, 4.67, and 5.25 hours after vonicog alfa injection (50 IU/kg). The 2 injections were given 1 month apart to avoid a cross-reaction. (C) FVIII/VWF:Ag and VWF:GPIbM/VWF:Ag ratios after injection of IVIG. (D) Normalized VWF:GPIbM/VWF:Ag ratio after injection of vonicog alfa, corresponding to the ratio for each time (t), of VWF:GPIbM(t) − VWF:GPIbM(t_0_) divided by VWF:Ag(t) − VWF:Ag(t_0_).

Two therapeutic options were considered: administration of IVIG or recombinant VWF concentrate, vonicog alfa (Veyvondi; Takeda Pharmaceuticals). After IVIG administration (1 g/kg), FVIII and VWF:Ag levels doubled, while VWF:GPIbM remained stable at around 30 to 40 IU/dL during a 14-day follow-up (Figure 2A). Administration of vonicog alfa (50 IU/kg) resulted in a 2-fold increase in VWF:Ag levels and a 6-fold increase (25-150 IU/dL) in VWF:GPIbM levels, which remained above 100 IU/dL for at least 5.25 hours (Figure 2B). The calculated half-life of vonicog alfa in this patient was 5.5 hours, while the theoretical half-life is 19 hours. The ratio of FVIII/VWF:Ag and the ratio of VWF:GPIbM/VWF:Ag both remained stable at 1.1 and 0.3, respectively, after IVIG administration (Figure 2C). In order to study more precisely the evolution of VWF:Ac and VWF:Ag in relation to vonicog alfa administered alone to the patient, it is necessary to subtract from the total VWF (exogenous VWF from vonicog alfa added to the patient’s endogenous VWF) the patient's VWF corresponding to the VWF measured at t_0_ before injection. The differences of VWF:Ag(t) − VWF:Ag(t_0_) and VWF:GPIbM(t) − VWF:GPIbM(t_0_) represent VWF:Ag and VWF:Ac, respectively, in relation to vonicog alfa alone at time t. Consequently, the normalized VWF:GPIbM/VWF:Ag ratio (Figure 2D), corresponding to each time (t), to VWF:GPIbM(t) − VWF:GPIbM(t_0_) divided by VWF:Ag(t) − VWF:Ag(t_0_) represents the Ac/Ag ratio in relation to vonicog alfa alone. This ratio also indirectly assesses the degree of multimerization of the exogenous VWF administered to the patient, as the GPIbM activity assay is quite sensitive to the degree of VWF multimerization.

## 3 Discussion

A literature review identified only 2 case reports of type 2B AVWS. The first one by Karger et al. [14] in 2011 described a 34-year-old patient admitted to the gastroenterology department for surgical intervention. He had a history of prolonged bleeding after a dental extraction 1 year before. The biological explorations revealed a monoclonal IgG kappa, a FVIII at 16 IU/dL, and VWF:GPIbM at 6 IU/dL. RIPA at 0.5 mg/mL showed 70% aggregation. The surgery was supervised, and desmopressin was administered without causing significant thrombocytopenia (reaching a minimum of 136 × 10^9^/L). Subsequently, FVIII/VWF concentrate (Haemate P, CSL Behring) was administered but proved ineffective, leading to the need for rFVIIa treatment. The second, reported by Scepanskya et al. [15] in 2014, was a 61-year-old patient with a significant bleeding history since age 54. The serum protein electrophoresis showed a monoclonal IgG quantified at 12 g/L. RIPA could not be performed due to platelet aggregates, but the 2B AVWS was proved by paradoxical aggregation at low doses of ristocetin when patient plasma and control platelets were mixed. During treatment, the patient experienced multiple bleeding episodes treated by Haemate P and rFVIIa. The patient was treated with IVIG, which proved successful in increasing the VWF:GPIbM level from 34 to 87 IU/dL.

Our patient’s clinical and biological presentation mimicked a VWD2B. Nevertheless, the acquired nature of the disease was indisputable, given the absence of bleeding complications in the past surgeries and the absence of mutations in the *VWF* or *GP1BA* genes, leading to eliminate constitutional VWD or pseudo-VWD. The low VWF:GPIb/VWF:Ag ratio also made it possible to exclude a false 2B VWD, as described by Favaloro et al. [16]. This case showed that low doses of ristocetin-induced paradoxical aggregation, which was caused by a thermostable component. This conclusion was drawn after observing that using heated patient PPP mixed with control PRP resulted in positive RIPA (ristocetin at 0.5 mg/mL), while the heated control PPP mixed with control PRP resulted in negative RIPA under similar conditions. This experiment suggests that the thermostable component of the patient’s plasma, which is stable at 58 °C (similar to antibodies), leads to the type 2B VWD phenomenon in the control PRP. The thermostable component was identified as an IgG since the paradoxical aggregation of RIPA at a low dose of ristocetin was significantly reduced after the depletion of IgG from the patient’s plasma. There is a high probability that the thermostable component is the patient’s monoclonal IgG lambda.

In contrast, the therapeutic tests with IVIG and vonicog alfa resulted in markedly different outcomes. The IVIG test resulted in an increase in FVIII, VWF:Ag, and VWF:Ac levels, while the ratios of FVIII/VWF:Ag and VWF:Ac/VWF:Ag remained constant. It is possible to speculate on a dual VWF clearance mechanism. The first mechanism might be quantitative and performed by macrophages, related to a monoclonal IgG binding to the patient’s VWF. The second mechanism could be qualitative and related to the clearance of HMW multimers due to increased VWF affinity to platelet GPIb. The increase in FVIII and VWF (VWF:Ag and VWF:Ac) during the IVIG test indicates the possibility of IVIG administration inhibiting the quantitative clearance mechanism by macrophages observed in most type 1 AVWS during myeloma, despite the normal VWF:pp/VWF:Ag ratio. The VWF:pp/VWF:Ag ratio is only increased in 55% of monoclonal gammopathies inducing type 1 AVWS. Therefore, such a clearance mechanism cannot be formally ruled out even if the VWF:pp/VWF:Ag ratio is normal [17].

The steady ratios of FVIII/VWF:Ag and VWF:Ac/VWF:Ag suggest that the mechanism of qualitative clearance by platelets is not affected by IVIG. The normalized VWF:GPIbM/VWF:Ag ratio evaluates the VWF:GPIbM/VWF:Ag ratio related to vonicog alfa alone and implicitly measures the level of exogenous VWF multimerization given to the patient. After vonicog alfa administration, the normalized ratio value was almost 1, whereas, in non-AVWS patients, it is typically 1.5 to 2, indicating an extensive multimerization of vonicog alfa. Hence, the relatively lower value of the normalized ratio to 1 in our patient is likely due to the rapid depletion of HMW vonicog alfa multimers following administration. A possible mechanism could be the binding of monoclonal IgG to vonicog alfa, resulting in increased affinity of vonicog alfa for platelets and rapid clearance of HMW multimers by platelets. Additionally, there was a gradual decrease in the normalized ratio over time, indicating the gradual proteolysis of vonicog alfa by ADAMTS-13 over time on top of the already established rapid clearance mechanism, although we cannot rule out a complementary and slower clearance of HMW VWF by platelets. The absence of a reliable platelet count does not allow us to draw any definitive conclusions. These 2 clearance mechanisms may explain the 3.5-fold shortened half-life of vonicog alfa.

## 4 Conclusion

We report the third case of type 2B AVWS and demonstrate that this type 2B AVWS resulted from a monoclonal IgG. Recombinant VWF concentrate treatment by vonicog alfa was effective in our case. Given the apparent ineffectiveness of immunosuppressive treatments (rituximab, cyclophosphamide, and azathioprine) in non-IgM monoclonal gammopathy of undetermined significance, alternative approaches may have been considered, particularly in the context of a higher risk of hemorrhage [18], which is not the case for our patient. One potential alternative is plasma exchange. Early treatment in MGUS with myeloma therapies (bortezomib, thalidomide, lenalidomide, daratumumab, etc.) is a potential avenue for further investigation. However, these latter therapies carry infectious risks and are more complex and costly to implement.
