# Supplementary material for: A rare case of acquired von Willebrand syndrome type 2B: diagnosis, treatment, and underlying pathophysiology
Source: Res Pract Thromb Haemost. 2024 Jul 19;8(5):102516. doi: 10.1016/j.rpth.2024.102516 (PMC11369413; doi:10.1016/j.rpth.2024.102516)
Supplement: Supplementary Figure [file mmc1.pdf]

SUPPLEMENTARY MATERIAL

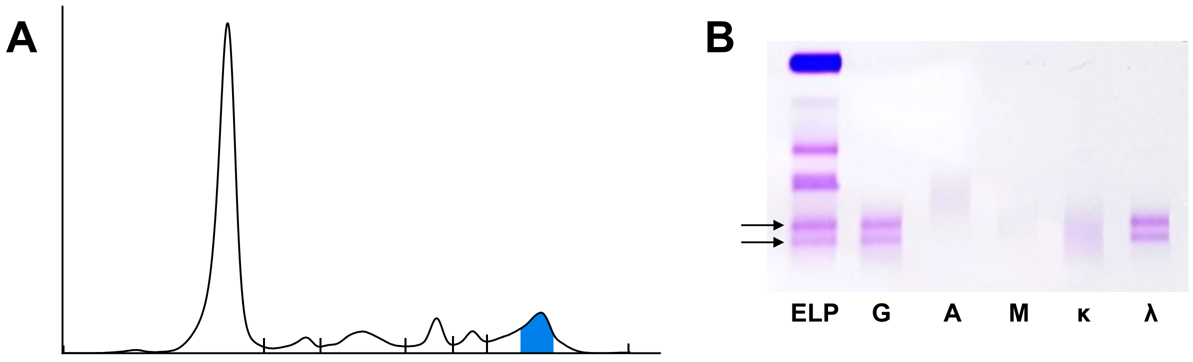

Figure: **Monoclonal immunoglobulin characterization.** (A) Patient serum protein electrophoresis, monoclonal immunoglobulin G lambda appeared in blue. (B) Patient serum immunofixation electrophoresis, arrows show the two monoclonal IgG lambda.

Abbreviations: ELP, serum protein electrophoresis; G, immunoglobulin G; A, immunoglobulin A; M, immunoglobulin M; κ, kappa light chain; λ, lambda light chain.
